# Identification of epidemic scarlet fever group A *Streptococcus* strains in the paediatric population of Houston, TX, USA

**DOI:** 10.1099/acmi.0.000274

**Published:** 2021-10-26

**Authors:** M. Belen Cubria, Jose Delgado, Brittany J. Shah, Misu A. Sanson, Anthony R. Flores

**Affiliations:** ^1^​ Division of Infectious Diseases, Department of Pediatrics, McGovern Medical School at the University of Texas Health Sciences Center at Houston, Houston, Texas, USA; ^2^​ Center for Antimicrobial Resistance and Microbial Genomics, McGovern Medical School at the University of Texas Health Sciences Center at Houston, Houston, Texas, USA

**Keywords:** antimicrobial resistance, *emm12*, group A *Streptococcus*, paediatric, scarlet fever

## Abstract

Scarlet fever (SF) has recently been associated with group A streptococcal (GAS) strains possessing multidrug resistance and specific streptococcal exotoxins. We screened a local surveillance collection of GAS *emm12* strains in Houston, TX, USA for antimicrobial resistance and identified a single isolate matching the antimicrobial resistance pattern previously reported for SF clones. Using whole-genome sequencing and combining genome sequence data derived from national surveillance databases, we identified additional *emm12* GAS clones similar to those associated with prior SF outbreaks, emphasizing the need for continued surveillance for epidemic emergence in the USA.

## Data summary

All raw genome sequences generated in this study are publicly available from the National Center for Biotechnology Information (NCBI) under BioProject accession number PRJNA608028. In addition, sequences from BioProjects PRJNA471864, PRJNA395240, PRJNA416675, PRJEB2675 and PRJNA13551 were used in comparative phylogenomics analysis, as indicated in Tables 1 and S1 (available in the online version of this article).

**Table 1.** Group A streptococcal strains used in this study

Region	Year(s)	*n*	Accession no.*
Houston	2013–2018	22	PRJNA608028
CDC ABCs (USA)†	2015–2017	408	PRJNA395240
Hong Kong/PR China	2011	177	PRJNA416675/PRJEB2657
UK	2014	63	PRJEB13551

*BioProject number at National Centr for Biotechnology Information (NCBI). Detailed strain information provided in Table S1.

†Centers for Disease Control and Prevention (CDC), Active Bacterial Core Surveillance (ABCs) includes 10 regions in the USA and a catchment area of approximately 44 million people (https://www.cdc.gov/abcs/index.html).

## Introduction

Epidemic scarlet fever (SF) caused by group A *Streptococcus* (GAS) was an important cause of morbidity and mortality in children during the 19th and early 20th centuries [1]. However, in 2011, an upsurge in the number of scarlet fever notifications was reported in Hong Kong, Beijing, Vietnam and the Republic of Korea [2]. The majority of isolates characterized from outbreaks in Hong Kong and mainland China were found to be of *emm* type 12 (*emm12*) and harbour two mobile genetic elements (MGE): an integrative and conjugative element (ICE-*emm12*) encoding tetracycline and macrolide resistance and a prophage (ϕHKU.vir) encoding two exotoxins (SSA and SpeC) and the DNase Sda1 [3, 4]. Subsequent outbreaks have since been noted in the UK and also include the epidemic *emm12* SF clone [5, 6]. MGE associated with *emm12* SF outbreaks have been identified in additional *emm* types, including *emm1* GAS strains [7]. Several factors have been proposed to play a role in triggering recent SF outbreaks, including enhanced capacity of GAS to cause SF through introduction of genetic elements, changes in host immunity, co-infection with an as yet unidentified pathogen and environmental factors [2, 8]. Most recently, studies suggest that the ϕHKU.vir-encoded exotoxins (SpeC and Spd1) act together in the *emm12* background to enhance colonization in a mouse model [9]. However, the precise underlying cause of these outbreaks remains uncertain. Re-examination of local and national GAS surveillance databases for the presence of epidemic SF-associated *emm12* clones is warranted, given their persistence in multiple outbreaks.

We have conducted GAS disease strain surveillance in the Texas Medical Center in Houston, TX, USA since 2013. Our GAS strain surveillance is unique in that we collect isolates derived from multiple disease states, including invasive, skin and soft tissue, and pharyngeal infections [10]. We sought to examine our collection for the presence of SF epidemic-associated *emm12* isolates. Molecular typing of GAS isolates from January 2013–July 2018 was performed to determine *emm* type. A total 300 *emm12* GAS isolates were identified and underwent antimicrobial susceptibility testing to tetracycline and erythromycin using disc diffusion [11]. Whole-genome sequencing was performed on a subset of *emm12* isolates (*n*=22) and any isolate resistant to both erythromycin and tetracycline using an Illumina MiSeq (300 bp PE). Raw sequencing reads were assembled using SPAdes and queried for the presence of exotoxin, DNase and resistance genes using a custom algorithm as previously described [11]. Complete genomes were resolved using long-read sequencing on an Oxford Nanopore MinION instrument and assembled as previously described [11]. Phylogenetic reconstruction based on biallelic single-nucleotide polymorphisms (SNPs) relative to the reference strain MGAS2096 was performed and compared to the subset of Houston *emm12* strains (*n*=22) and previously sequenced *emm12* GAS strains from the Centers for Disease Control and Prevention (CDC) Active Bacterial Core Surveillance (ABCs) (2015–2017, *n*=408), Hong Kong/PR China (2011, *n*=177) and the UK (2014, *n*=63) (see Table 1).

**Fig. 1.**
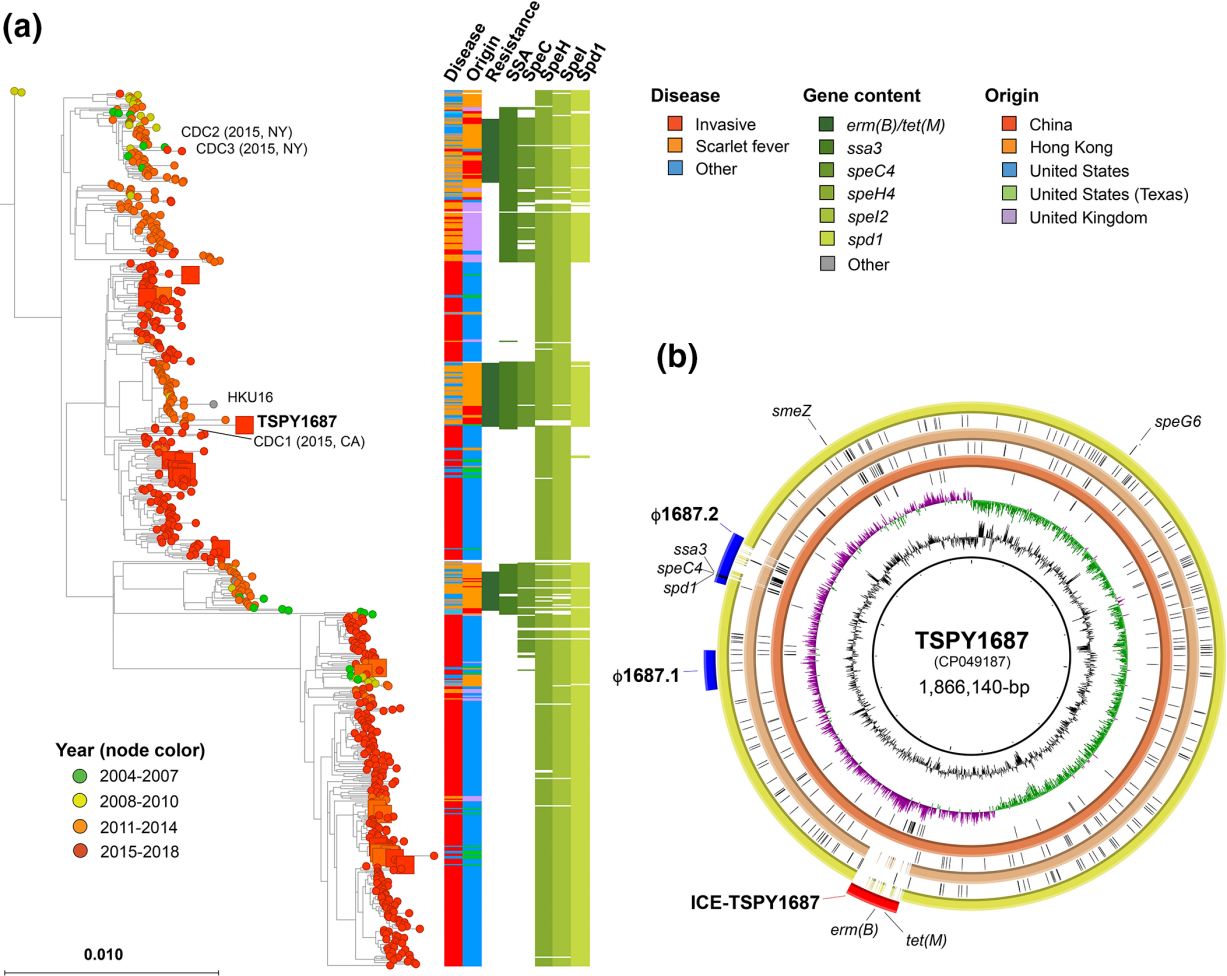
**(a**) Comparison of Houston/US SF-like *emm12* GAS strains to previously described SF outbreak strains. Phylogenetic reconstruction of 670 *emm12* GAS strains (Table 1) based on 7159 core genome biallelic SNP loci relative to the reference strain MGAS2096 using RAxML. Disease type, country, resistance genotype and exotoxin/DNase content are indicated in the vertical bars on the right. Strains mentioned in the text are labelled. (**b**) Comparison of TSPY1687 to reference *emm12* GAS genomes. The complete genome of TSPY1687 was resolved using a combination of short- and long-read sequences and annotated (PGAP at NCBI). blast comparisons to HKU16 (AFRY01000001), SP1336 (CP031738) and MGAS2096 (CP000261) are indicated in red, orange and yellow rings (inside to outside), respectively. Ring legend (inside to outside): (1) GC content, (2) GC skew, (3) SNPs relative to HKU16, (4) HKU16 blast comparison, (5) SNPs relative to SP1336, (6) SP1336 blast comparison, (7) SNPs relative to MGAS2096, (8) MGAS2096 blast comparison, (9) genomic features of TSPY1687 including prophage (blue), ICE (red) and exotoxin/DNase genes (black).

From the 300 *emm12* GAS isolates from Houston, TX screened for antimicrobial resistance, a total of 14 (5 %) were identified with resistance to erythromycin or tetracycline and a single isolate (TSPY1687 – pharyngitis from 2018) was identified to be resistant to both antibiotics. Consistent with previous reports on GAS [12], we saw complete congruence between resistance phenotype and genotype for all Houston, TX strains analysed. For comparison, we also queried the genomes of all *emm12* GAS isolates sequenced by the CDC ABCs and discovered three additional isolates with erythromycin and tetracycline resistance genes (CDC1–3, Fig. 1a). Phylogenetic analysis of geographically and temporally diverse *emm12* strains (*n*=670; Table S1) demonstrated a close relationship of all four US isolates to groups of strains from previous SF outbreaks in Hong Kong and PR China (Fig. 1a). Strains originating from Houston, TX showed a similar distribution to that of the ABCs (Fig. 1a) and consistent with previously observed surveillance studies [11]. Moreover, strains originating from the USA showed the presence of *ssa* – an exotoxin gene associated with previous SF outbreaks in Asia [3, 4]. Consistent with phenotypic testing, genome sequence analysis of Houston and CDC ABCs isolates showed a relative lack of resistance genes. Of the US strains exhibiting resistance to erythromycin and tetracycline, CDC1 (isolated in 2015 in California) was phylogenetically very similar to both HKU16 and TSPY1687 (Fig. 1a).

**Fig. 2.**
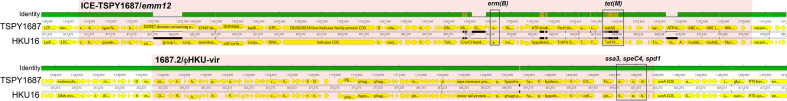
Comparison of exotoxin and antimicrobial resistance encoding MGE between TSPY1687 and HKU16. progressiveMauve alignments of: (top alignment) ICE-TSPY1687 (TSPY1687) compared to ICE-*emm12* (HKU16) with antimicrobial resistance genes boxed and labelled and (bottom alignment) 1687.2 (TSPY1687) relative to ϕHKU.vir (HKU16) with exotoxin genes boxed and labelled. Bar at the top of each alignment indicates degree of homology: green (100%), yellow (>30 %), none (absent in one genome). Additionally, black markings on solid bar below annotations indicate nucleotide differences.

Using a combination of short- and long-read genome sequences, we completely resolved the genome of TSPY1687, the first described SF-like epidemic clone in the USA. We compared TSPY1687 to the benchmark Hong Kong SF strain, HKU16, and confirmed the presence of both ICE-*emm12* and ϕHKU.vir (Fig. 1b). Only 58 SNPs within the core (excluding MGE) differentiated TSPY1687 from HKU16. Among the identified MGE, TSPY1687 possessed two prophage, ICE-*emm12* with *erm(B*) and *tet(M),* and differed by a single prophage that was devoid of any exotoxin or DNase genes (Fig. 1b). The ϕHKU.vir (1687.2, in Fig. 1b) and resistance gene-encoding ICE (ICE-TSPY1687, in Fig. 1b) showed a high degree of similarity at the nucleotide level and in terms of gene content (Fig. 2).

## Conclusions

A rapid rise in the incidence of SF in the Republic of Korea, PR China and Hong Kong marked the beginning of an epidemic in Asia. Extensive surveillance identified GAS strains of primarily *emm*12 (76.4 %) and *emm*1 (17.1 %) lineages [4]. Outbreaks have subsequently been noted in the UK with *emm12* GAS strains similar to the original outbreak strains in Asia [5]. Most recently, *emm12* GAS strains more closely related to those from the UK were identified in Australia [8]. We have identified the first *emm12* SF-like clone in the USA using our local GAS strain surveillance and shown additional clones circulating within the US population using publicly available national surveillance databases. SF is a notifiable disease in many Asian countries and the UK but not the USA. Interestingly, none of the four *emm12* SF-like clones identified in our study were derived from SF cases. TSPY1687 was isolated following a case of pharyngitis in an 8-year-old child in 2018 but lacked rash or other symptoms (e.g. strawberry tongue) associated with SF. Likewise, the three CDC ABCs *emm12* SF-like strains were from cases of invasive disease in 2015 but at opposite ends of the country (New York and California). Unlike in our local surveillance, we are unable to further query electronic medical records for additional signs and symptoms in the CDC ABCs cases. Inasmuch as GAS whole-genome sequencing analyses have shown that invasive strains are derived from the population of pharyngeal strains [13], our findings suggest a possible greater presence of *emm12* SF clones in the US population. At this time, it is unclear if more robust prospective surveillance for GAS pharyngitis would identify additional cases in the USA. Alternatively, it is also possible that the small number of *emm12* SF-like clones identified in our analysis represent individual importation events from outbreak regions. Importantly, our data confirm a worldwide spread of *emm12* SF clones and suggest that additional factors apart from GAS strain contribute to the development of SF. In summary, our findings emphasize the role of GAS surveillance, antimicrobial susceptibility profiling and heightened awareness of possible SF in Houston and the USA.

## Supplementary Data

Supplementary material 2
